# Strategies for Heterogeneous R&D Alliances of In Vitro Diagnostics Firms in Rapidly Catching-Up Economies

**DOI:** 10.3390/ijerph17103688

**Published:** 2020-05-23

**Authors:** Chi-Yo Huang, I-Ling Tung

**Affiliations:** Department of Industrial Education, National Taiwan Normal University, Taipei 10610, Taiwan; iristung@abnova.com.tw

**Keywords:** in vitro diagnostics (IVD) industry, heterogeneous alliance, Decision-Making Trial and Evaluation Laboratory (DEMATEL), DEMATEL-based analytic network process (DANP), VlseKriterijumska Optimizacija I Kompromisno Resenje (VIKOR)

## Abstract

Most developed countries already have high-quality in vitro diagnostic (IVD) techniques for diseases, but developing countries often do not have access to these technologies and cannot afford them. Enabling firms to leverage external resources to optimize their research and development (R&D) performance has become one of the most critical issues for small and medium-sized late-coming IVD firms. R&D alliances, especially heterogeneous alliances, are necessary for releasing the resource limitations of late-coming small and medium-sized enterprises (SMEs) and reaching the metaoptimum of the R&D performances. However, to the authors’ knowledge, a few, if any, previous studies have investigated the key success factors and strategies of heterogeneous alliances in the IVD industry. Therefore, the authors aim to define the critical factors for evaluating and selecting strategies for heterogeneous alliances in the IVD industry. A Decision-Making Trial and Evaluation Laboratory (DEMATEL)-based analytic network process (DANP) was proposed to prioritize the weights associated with the evaluation criteria. Then, a heterogeneous R&D alliance strategy was derived from the compromise ranking based on the modified VlseKriterijumska Optimizacija I Kompromisno Resenje (VIKOR) method. An empirical study of major Taiwanese IVD firms’ evaluation and selection of heterogeneous R&D alliance strategies will be used to reveal the practicability of the analytic framework. Based on the analytic results, the joint venture strategy is the most suitable heterogeneous R&D alliance strategy for IVD firms in rapidly catching-up economies. These results can serve as the basis for heterogeneous R&D alliance strategy definitions in the IVD industry in the future.

## 1. Introduction and Background

In vitro diagnostic (IVD) devices are the reagents, instruments, and systems used for the diagnosis of diseases or other medical conditions; such devices are different from general medical devices and mainly rely on nondirect contact. Commercial IVD devices can be used to diagnose many infectious diseases. In addition to off-the-shelf IVD medical devices, laboratories can assemble and use internal tests of their own. For many diseases, including human immunodeficiency virus (HIV) infection, whether a patient can receive appropriate treatment depends on the availability of appropriate diagnostic tests. Because IVD equipment can help medical staff to diagnose patients more accurately; it has been widely used in all levels of health care systems in recent years [1].

The IVD industry includes technology-intensive companies in the pharmaceutical and medicine manufacturing industries. Although generally considered an extension of the pharmaceutical industry, the IVD industry is strongly related to the frontiers of human and animal disease diagnosis, involving a wide range of genetic research and providing participants with a wealth of technical and knowledge-intensive resources [2]. Companies in this industry produce chemical, biological, or even radioactive materials for the diagnosis or monitoring of several diseases in body fluids or tissues. For example, Alliance Pharmaceuticals, the industry leader, developed and manufactured “Imagent,” an injectable ultrasound contrast agent that can be used in conjunction with echocardiography for the diagnosis of heart disease. Therefore, technical resources are the core competences for competitions in the industry [2]. Both large companies and small and medium-sized enterprises (SMEs) in the IVD industry have been urged to pursue new technologies and their commercialization to fulfill market needs and maximize profits.

Biotechnology, in general, and IVD, in particular, are supersets of technologies that may be functional for many industries, such as molecular biology, genomics, medicine, agriculture, chemicals, food, and environmental disposal [3]. In the IVD market, both governments and patients struggle to pay for high-quality products. However, the IVD market has grown quickly, encouraged by market demands and government policies. An increasing number of elderly people are susceptible to a variety of diseases and require regular diagnostic examinations, which is expected to have a significant impact on the market [4]. Greater awareness of health care, increased incidences of chronic and infectious diseases, and more cost-effective diagnostic solutions have also driven the market’s growth [5]. Similarly, usage of automatic instruments and field-care diagnostic solutions is rising, and government spending on health care has increased around the world, including in North America, Europe, Asia Pacific, Latin America, the Middle East, and Africa; thus, quicker and more accurate test results are anticipated to drive growth in the global IVD market [5]. In terms of cost-effectiveness, accuracy, and portability, new product launches and technology developments are anticipated to drive market growth during the forecast year [4]. BisReport. [4] predicted that the global IVD market, which was worth US $69.6 billion in 2018, would grow at a compound annual growth rate (CAGR) of 4.75% from 2019 to 2026. Hamelburg [5] made a similar forecast, predicting a market of $93.614 billion by 2025, with a CAGR of 4.8% from 2018 to 2025. North America is expected to be the largest market for the entire forecast period.

Because they are aiming for such a large market, both large companies and SMEs in the IVD industry are encouraged to pursue new technologies and their commercialization to fulfill market needs and maximize profits. In most developed countries, high-quality diagnostic techniques are widely used for diagnosing infectious diseases. However, in developing countries, these advanced technologies are neither available nor affordable, despite the fact that infectious diseases cause even more serious harm in these regions of the world. Although it is a long-term goal to establish the innovation capabilities of advanced diagnostic techniques in developing countries, technology transfer and the local production of such techniques can be a feasible, effective, and sustainable strategy in the near future [1].

According to the World Health Organization (WHO) [1], examples of successfully transferring diagnostic technologies from advanced countries to developing nation-states involve SMEs, multinational corporations, nonprofit organizations, and the public sector. An example of the successful transfer of novel up-to-date IVD technology is the transfer of techniques from IVD companies in developed countries to nonprofit public institutions in developing countries. Such technology transfer agreements allow for not only the local production of IVD-based technology but also its use for further research and development (R&D) to test for diseases prevalent in the specific nation-states transferring the technology. Despite these successful paradigms, there are still many obstacles to the technology transfer and local production of IVD techniques, including the lack of financial investments for R&D and the inability to support local markets for cost-effective manufacturing. Most IVD firms in late-coming economies are SMEs with very limited resources. Enabling firms to leverage external resources to optimize the R&D performance has become one of the most critical issues facing late-coming SMEs.

Sufficient resources are critical for the R&D of an IVD firm. For large companies in leading economies (e.g., Roche, Abbott, Siemens Healthineers, and Danaher) of the IVD industry, sufficient resources can support innovations through the required clinical trials. Scholars have already summarized that R&D alliances are the most important contributing factor to a company’s success in establishing innovation pipelines and commercializing inventions in the IVD industry. Such observations are especially true for rapidly catching-up SMEs with minimal resources.

R&D alliances have increasingly been formed in various industries, and especially in the IVD industry. However, the failures and problems associated with R&D alliances discourage managers of IVD firms. Typical failures and problems include disparate corporate cultures and divergent time frames for objectives. Such concerns are management challenges.

IVD is an industry characterized as having knowledge workers, long R&D cycles, huge capital expenditures, and high levels of uncertainty. Nonetheless, most IVD firms in late-coming economies are SMEs with very limited resources. It is almost impossible for these SMEs to obtain competencies from other technical domains, such as artificial intelligence (A.I.) or telecommunications, which are needed for the R&D of some IVD devices. However, it is especially important that such SMEs have successful and efficient R&D. Alliances such as various types of license shares, technology transfers, and R&D joint ventures [6] are typical approaches for SMEs to leverage outside resources and achieve successful innovations. Thus, determining how firms can leverage external resources to optimize R&D performance has become a critical issue for late-coming SMEs. R&D alliances, in general, and heterogeneous alliances, in particular, are essential for overcoming the resource limitations of late-coming SMEs and reaching the metaoptimum of R&D performances. A typical example of successful heterogeneous R&D alliances in the European Heterogeneous Technology Alliance is the integration of wireless body area networks (WBAN) with health and wellness applications. An example of success for SMEs from emerging economies includes the recent strategic alliance between the Taiwan-based Abnova and the Israel-based BioView. Both firms successfully formed an alliance in developing an all-in-one imaging system and A.I. solution for identifications of circulating tumor cells (CTCs) and circulating fetal cells (CFCs) [7]. In addition, the Taiwan-based PlexBio, a clinical cancer diagnostics developer, and the Japan-based Denka, a Japanese manufacturer of chemicals, developed a strategic partnership recently [8]. Heterogeneous R&D alliances have become particular targets for close collaborations and risk-sharing partnerships.

However, to the authors’ knowledge, a few, if any, existing studies have explored the critical factors and strategies of heterogeneous alliances in the IVD industry. Therefore, this study aims to derive the key success factors for such heterogeneous alliances. Hence, the research purposes of this study are threefold: (1) derive the key dimensions/criteria used to define R&D alliances’ strategies for SMEs in the IVD industry, (2) access and choose the most appropriate R&D alliance strategies for late-coming SMEs, and (3) present the practicability of the expert system-based analytic framework by comparing the outcomes based on judgments by experts from the IVD industry.

Consequently, this study seeks to derive the key success factors for such heterogeneous alliances. Possible factors will first be identified through a review of the existing literature, focusing predominantly on the theories of transaction cost, resource-based view, strategic behavior, resource dependence, and organizational learning. Candidates for the heterogeneous R&D alliance strategies will also be defined using the same approach. The decision problem will be structured by utilizing the Decision-Making Trial and Evaluation Laboratory (DEMATEL) method based on experts’ opinions. A causal relationship established through the influence relationships among aspects and criteria can thus be derived. Then, the weights associated with each aspect and criterion will be determined using the DEMATEL-based analytic network process (DANP). Finally, a heterogeneous R&D strategy can be derived from the compromise ranking calculated by introducing the derived weights and the performance scores versus each aspect and score using the modified VIKOR. An empirical study based on major Taiwanese IVD firms’ evaluation and selection of heterogeneous R&D alliance strategies will be used to present the practicability of the proposed analytic framework. The most suitable heterogeneous R&D alliance strategy selected can be adopted by IVD firms in developing countries. Meanwhile, the well-verified analytic framework can be adopted by managers and scholars of the biotechnology industry, in general, and the IVD industry, specifically, to evaluate and select appropriate R&D alliance strategies.

The remainder of the paper is organized as follows. Section 2 of this work reviews past works on motivations of R&D alliances, including the transaction cost theory, resource-based theory, strategic behavior theory, resource dependence theory, and organizational learning theory. Existing works on heterogeneous alliance strategies for R&D and types of R&D alliances are also reviewed. Finally, the analytic framework is defined. Section 3 introduces the methods, including the modified Delphi, DEMATEL, DANP, and VIKOR. Section 4 uses an empirical study based on a real case of a leading Taiwanese IVD firm’s adoption of the heterogeneous R&D alliance strategy to demonstrate the feasibility of the proposed analytic framework. Managerial implications, as well as advances in research methods, are presented in Section 5. Finally, the conclusion of the article is presented in Section 6.

## 2. Literature Review

This section introduces literature on the definition, dilemma, and types of the R&D alliances. The literature cites well-known R&D alliances’ motivation theories, including transaction cost theory, resource-based theory, strategic behavior theory, resource dependence theory, and organizational learning theory.

### 2.1. Motivations of R&D Alliances

Innovation in general, and heterogeneous R&D in particular, can be costly, time consuming, and full of uncertainties. Due to the limited resources, establishing a network to access collective knowledge is crucial for the success of IVD firms. The formation and mode selection of the R&D alliance is determined by the motivations behind forming the alliance. The following subsections present the relevant theories that serve as the basis for developing the theoretical model of this paper.

#### 2.1.1. Transaction Cost

Transaction costs refer to the cost of organizing information, coordinating behavior, protecting the benefits of the transacting parties, monitoring the transactions, inducing the appropriate behavior adjustments, etc. [9]. Suematsu [10] argued that a transaction cost is the cost generated when searching for a partner, gathering information, negotiating and reaching a decision, exchanging, as well as engaging in expost exchanges. In addition to purchase and sales activities between and within the firm, all communication and interactions within the firm are included from the aspect of transaction costs [10]. In general, transaction costs comprise all those costs not directly incurred in the physical process of production [11].

Transaction cost theory is an economic approach to analyze an organization; it concludes that the institutional structures (except markets) might be a better method of governing economic activities. In 1937, Coase [12] argued that firms and markets are alternate structures with different transaction costs. The costs include those before the transaction (e.g., costs required for drafting and negotiating contracts) and after the transaction (e.g., the audit and enforcement of agreements). Transaction cost theory points out that, due to the uncertainty of the environment and the limitation of human rationality, the transaction process will not be perfect; thus, transaction costs are inevitable [12].

In transaction cost economics, dimensions for describing transactions are (1) assets’ specificity, (2) uncertainty, and (3) frequency [13,14,15]. Transaction cost theory has been widely adopted in analyzing a number of different organizational behaviors [16,17]. Transaction cost theory emphasizes cost-saving thinking, and some researchers have pointed out that, as a result of the R&D alliance, firms can share the fixed costs of R&D activities, reduce probable risks, enter expected or possible markets, and achieve economies of scale to reduce violent competition and get government subsidies [18].

#### 2.1.2. Resource-Based Theory

A resource is any substantial or intellectual property available to a firm that enables the production and marketing of products or services valuable to specific segments in the target markets [19]. The complementarity of resources influences the success of alliance success indirectly through idiosyncratic resources [20]. Resources are meaningfully heterogeneous and, in some ways, unique between each company. The resource-based view (RBV) of the firm is a set of related theories sharing the assumptions of resource heterogeneity and resource immobility across firms [21]. From this aspect, a firm is a bundle of resources, capabilities, or routines that creates value and cannot be easily imitated or appropriated by competitors due to isolating mechanisms [21]. Resource-based theory largely focuses upon the importance of resource strengths [22]. Companies strategically balance the use of existing internal resources with the deployment of external resources to keep pace with growth [23].

From the R&D perspective, company resources refer to available tangible or intangible entities, which include capabilities, technologies, assets, patents, information, organizational processes, and human resources (knowledge and skills) that could enhance R&D efficiency and effectiveness [24,25,26]. Thus, an effective R&D alliance enables partners to integrate their resources successfully, which can contribute to improve R&D, thereby increasing competitive advantages.

#### 2.1.3. Strategic Behavior Theory

Strategic behavior theory is based on the assumption that competitive advantages result from resources that can impact a firm’s comparative advantage over other firms for a specific period [27]. According to strategic behavior theory, firms establish cooperative arrangements in order to maximize long-term profitability by enhancing their competitive position [28]. The strategic behavior theory proposed by Kogut [27] and Jarillo [29] argued that firms enter cooperative alliances due to long-standing strategic considerations, regardless of instant concerns regarding costs and benefits [30]. In other words, strategic behavior theory predicts that firms will engage in cooperative activities as a means of achieving overall strategic objectives regardless of their effect on specific transaction costs [28]. Although competing theories (e.g., transaction cost theory and resource dependence theory) argue that the evaluation and selection of a suitable organizational behavior is based on either a desire to minimize transaction costs or the need to offset the dependencies on external resources, strategic behavior theory argues that such adaptation behaviors are the consequences of strategic goals [27,28,31].

#### 2.1.4. Resource Dependence Theory

The resource dependence theory proposed by Pfeffer and Salancik [32] characterizes a firm as an open system reliant on the conditions of the external environment. The theory has already been widely adopted in the analysis of organizational behavior and strategic management. The main principles of the theory are threefold: (1) the prominence of the organization’s capabilities to obtain and sustain (tangible and intangible) resources required for survival; (2) the organization’s existence in the organizational network, which affects access to the mandatory resources; and (3) companies’ attempts to decrease their dependencies on other organizations (regarding access to resources) while trying to make other organizations more dependent on them [33,34]. When limited by the external environment, organizations attempt to reduce the dependency on the environment and any uncertainty by at least partially developing environmental strategies [33,34].

The resource dependence theory has been widely applied in explaining how organizations can minimize environmental interdependence and uncertainty across the research domains [35]. According to Nienhüser [36], three major discrepancies exist between the resource dependence theory and the resource-based view. First, the resource dependence theory focuses more on the external perspective, whereas the resource-based view focuses more on the internal perspective [36]. Second, the theoretical background used to develop explanations differs [36]. Finally, the resource dependence theory is a more expressive, explicit, and value-neutral method, whereas the resource-based view is understandably more prescriptive because it has been developed and embedded in the discourse environment of strategic management [36].

#### 2.1.5. Organizational Learning Theory

Organizational learning includes the acquisition, distribution, interpretation, and storage of knowledge by an organization [37]. The mobilization of organizational learning occurs through four types of knowledge conversion: (1) intuition, when the characteristics of learning at the individual level imply recognizing the patterns and/or possibilities generated by individual experiences; (2) interpretation, when bridging between the personal and organizational levels consists of explaining a concept or an idea using terms or actions; (3) integration, when acting as a convergence point amongst groups and organizational levels means that individuals reach consensus and take actions, suggesting mutual adaptation; and (4) institutionalization, when the introduction of routines provides guidance to individuals within the organization at the organizational level [38]. As the uncertainty and complexity associated with the technology increase, the transfer of knowledge becomes more and more important as firms are dependent on the process of open innovation to ensure longstanding competitiveness [39,40]. Learning from the counterpart of an alliance is fundamentally intended to acquire information and knowledge [41,42].

Knowledge is one of the most dominant strategic resources for competition. An R&D alliance is regarded as a major way to access external knowledge across firm boundaries and be realized as an opportunity for learning and increasing experience. However, another problem is that technologies’ interdependence might enhance both knowledge acquisition and leakage. Nowadays, the advancement of information technology has enabled firms to access knowledge from external partners when such knowledge has already become a major source of innovation. Therefore, R&D alliances have been widely adopted, as evident in the US pharmaceutical industry. The advantages of scale associated with R&D alliances can assist firms in commercializing best-selling drugs, taking technology risks, accelerating clinical experiments, and increasing the interests of alliance counterparts to license technology [43]. A good R&D alliance is designed to reduce concerns about leaking knowledge and, thus, encourage anticipated knowledge transfers in R&D alliances. As a result of interorganizational collaborations between the focal firm and the alliance partners, a more open and interactive R&D alliance is essential [43,44].

### 2.2. Heterogeneous Alliance Strategies for R&D

Strategic alliances are beneficial for realizing or ensuring competitive advantages, especially for small firms [45]. Firms in development can build strategic alliances with superior ones as a means to share or acquire knowledge from these alliance participants to transform into innovative and competitive ones [46]. There have been wide-ranging usages and various motivations for strategic alliances by the SMEs in the biotechnology industry [47]. Although participating alliances may require a firm to give up some autonomy, the firm can acquire know-how much faster than through internal R&D [47].

A heterogeneous alliance involves partners with different core competences [48]. From the viewpoint of a focal firm, alliance partners are located in various parts of the supply chain, including upstream, downstream, and horizontal organizations, and have their own various resource sets. Unlike a homogenous alliance, where partners’ resource sets are similar, a heterogeneous alliance allows a focal firm to exploit complementary resources within the alliance [49]. According to [50], heterogeneous alliance strategies for R&D can further be classified as the R&D alliance with competitors (horizontal), with suppliers or customers (vertical), and with universities and research institutes (“institutional” cooperation).

Most IVD firms in developing countries are SMEs. To face the severe global competition and operation pressure, developing potential new products efficiently and successfully for the developing economies is essential. Numerous studies have regarded the alliances as the critical factor for the continued existence and development of biotechnology firms [51,52,53,54]. In recent years, cross-industry alliances or interindustry collaborations have been formed to pursue the transformation of the current biotechnology industry.

#### 2.2.1. Definition of R&D Alliances

Innovation within a firm depends upon the ability to integrate competences. A firm may develop a new product in house or actively participate in an R&D alliance [55]. An R&D alliance refers to voluntary arrangements among firms or organizations that exchange, share, or jointly develop products or technologies [56].

An R&D alliance is a strategic alliance that aims to engage in R&D activities. The alliance consists of at least two parties, which can be firms, schools, R&D institutes, or governmental institutions; the parties do not control the ownership of each other’s technology. The major purpose of R&D alliances is to improve the technology level, innovate new products, and encourage intended new or complementary knowledge and technology learning. Tyler and Caner [57] explained that R&D alliances are formed for a variety of reasons, such as to access technological resources and capabilities, share R&D risks and costs, and explore new technological domains. Firms considering entering into an R&D alliance expect to improve their core competence while reducing risks of investment and environmental uncertainties. As the size of these firms is an important consideration or as the firms focus more on expanding core competences, the number and intensity of R&D alliances increase dramatically. The alliance networks enable firms to access various benefits that promote innovation. R&D alliances aim to access novel and complementary technologies and to accelerate the innovation and learning processes [58]. R&D alliances are more appropriate than other kinds of partnerships for achieving these goals. However, the R&D alliance is a complement instead of a substitute for a firm’s internal R&D [59,60,61]. Thus, R&D cooperation in general, and R&D alliances in particular, has become a core aspect of the innovation strategy of R&D-performing organizations over the last three decades [62].

To share alliance R&D results, the alliance participants cooperate through coinvestment and by sharing experts, technology, and resources to enhance the R&D process [63,64,65,66,67,68]. An R&D alliance shares well-matched goals, seeks common benefits, and allows for mutual dependence [69,70]. Optimal governance of R&D alliances is critical for obtaining the benefits of cooperation while reducing the risks [71].

#### 2.2.2. The Dilemma of R&D Alliances

The R&D alliance is a crucial solution for initiating innovations and enhancing companies’ performance. Despite the growing number and increasing significance of R&D alliances, many fail or perform poorly, indicating that R&D alliances are difficult to implement [72]. Each organization attempts to pursue the maximum benefits for itself beyond its partners’ interests. Collaboration does not always provide opportunities to internalize a partner’s skills; there may be a psychological barrier between alliance partners for fear that one party to the alliance may overlearn or the other side may have lost its skills [73]. No organization is able to integrate all capabilities required to commercialize technology and compete successfully in a wide variety of evolving fields of technology [74]. According to Rothaermel and Deeds [75], R&D alliances are full of high uncertainty, ambiguity, complexity, and the transfer of implicit knowledge. Thus, strong alliance management capabilities are required. The emphasis is on identifying the key factors of R&D alliances that small and medium-sized IVD companies can accept and what their concerns with R&D alliances are. With such information, R&D alliances can move forward.

### 2.3. Types of R&D Alliances

Various types of R&D alliances exist based on past works. Interorganizational relationships involve strategic alliances, joint ventures, agreements, licensing, cobranding, franchising, cross-sector partnerships, networks, trade associations, and consortia [76]. The crucial issues to be considered in the planning and governing of an alliance include (1) equity distribution, (2) term of agreement, and (3) relationship management [45]. The partners’ insights into risk, namely, uncertainty about the external environment’s changes, trust, and level of profit-sharing, reflect the factors that lead to the alliance’s efforts [77]. Each method for forming an R&D alliance can be explained as a firm’s arrangements to minimize all efforts necessary to develop successful products. The frequently observed governance structures of strategic alliances include (a) equity-based alliances, where the distribution of equity in a new alliance is mutually dominated by the parties being involved in R&D activities and (b) nonequity-based alliances, which include the codevelopment of technology between two or more parties in the project of R&D cooperation [18,78,79].

Low to medium technology level firms tend to adopt equity-based alliances. When there is a high possibility that a resource may accidentally be infringed upon, the firm tends to adopt nonequity-based alliances [18,56,80]. In contrast, to pursue the transfer of intangible assets, nonequity-based alliances are preferred by firms that contribute such implicit expertise [81]. Various forms of partnerships exist, including cooperation with customers, suppliers, competitors, universities, and research centers [82]. Nonequity-based alliances have been more attractive than equity-based alliances since the late 1980s for several reasons: (1) the higher cost of establishment, (2) the higher risk of intellectual property being infringed, and (3) the inconsistent goals among alliance members. According to the literature reviewed, high-tech firms tend to use nonequity-based alliances (such as contractual agreements, including technology and patent licensing, sharing, and joint R&D projects).

Normally, IVD firms deal with three categories of partners related to the supply chain: (1) upstream alliances that include research or higher education institutions, (2) horizontal alliances with other IVD companies, and (3) downstream alliances with established pharmaceutical companies (e.g., Roche and OSI collaborated with Abbott’s diagnostics division to develop a pharmacogenomic test to identify patients more likely to respond to the cancer drug Tarceva [83]). Different categories of alliances require various management capabilities within alliances [75,84]. The identification and selection of a suitable alliance type and partner(s) is also critical for the formation of an alliance.

The IVD industry is characterized as having knowledge workers, a long R&D cycle, large financial investments, and great uncertainty. Various factors motivate a firm to join heterogeneous R&D alliances, such as efficiency, capabilities, assets, and strategic purposes. The wide adoption of heterogeneous R&D alliances for innovations has become a dominant trend in the IVD industry [85]. Such alliances are formed based on the partners’ competences or intangible assets. Synergies of heterogeneous R&D alliances can also be developed based on partners’ complementary assets [85]. As only a small number of firms might individually accumulate all the competences required to develop a technology or product effectively to cover a wide variety of technologies [74], accepting heterogeneous R&D alliances has become the norm.

Based on the theoretical frameworks and models researchers have proposed that may motivate R&D alliances, the theoretical foundation of the current research will be proposed. Having reviewed the related theories, the evaluation criteria and rationality for selecting strategies for heterogeneous R&D alliances are, summarized in Table 1. The aspects and criteria will serve as the basis for developing the hybrid multiple criteria decision making (MCDM) analytical framework in this current study. The next section will define an analytic framework according to the literature reviewed. The analytic framework will be applied to derive the influences among constructs and criteria. The influential weights of each aspect and criterion will also be derived using the DANP. Finally, the most suitable strategies for IVD firms in the rapidly catching-up economies will be derived using the modified VIKOR method.

## 3. Analytic Framework and Methods

The evaluation and selection of suitable strategies for heterogeneous R&D alliances for an IVD firm in a rapidly catching-up economy are, instinctively, an MCDM problem. These factors include the motivations for a heterogeneous R&D alliance, such as the transaction cost, resource-based view, strategic behavior, and resource dependence and organizational learning theories. Therefore, a DEMATEL-based DANP-mV framework, based on the graph theory, can be used to construct the decision-making framework considering the influential relationships among all criteria. The proposed framework will be highly suitable for the selection of a strategy for heterogeneous R&D alliances for an IVD company.

**Figure 1:**
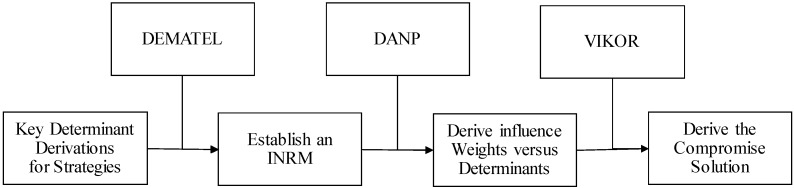
The flowchart of the decision-making framework.

To evaluate the relevance of heterogeneous R&D strategies for an IVD company, related aspects/criteria being summarized in Table 1 will be confirmed by experts using the modified Delphi method proposed by Murry and Hammons [110]. Then, the DEMATEL [111] technique is used to derive an influence relations map (IRM) and the interrelations between aspects and criteria. After that, the DANP proposed by Tzeng [112,113] will be used to derive the influence weights versus aspects and criteria based on the basic concepts of the analytic network process (ANP) being proposed by Saaty [114]. The VIKOR method [115] will be used to derive the compromise solution by introducing the influence weights being derived by using the DANP. This hybrid MCDM model is illustrated in Figure 1. The model has been used to resolve various decision-making problems such as brand evaluation [116], materials for engineering designs [117], low carbon suppliers [118], RFID technology evaluation and selection [119], and disaster-recovery site evaluation, selection, and improvement [120]. In summary, this evaluation framework consists of six main phases: (1) define the heterogeneous R&D alliance strategies and criteria through the literature review, (2) filter out the important criteria using the modified Delphi method, (3) construct the IRM among criteria using DEMATEL, (4) use DANP to derive the influence weights associated with each aspect and criterion, (5) sort the heterogeneous R&D alliance strategies and rank the priorities with VIKOR, and (6) decide on the type of heterogeneous R&D alliance strategies by analyzing the research results. These methods are discussed in the following subsections.

### 3.1. Modified Delphi Method

The Delphi approach was designed by Dalkey and Helmer [121] to gather and conclude experts’ views on particular issues. Murry and Hammons [110] improved the original Delphi method by substituting the traditional open survey with a carefully selected set of questionnaires. These questionnaires could be gathered from a variety of sources, including comprehensive results from past works, expert opinions, etc. Key benefits of the improved Delphi approach include: (1) increasing the number of replies from traditional Delphi methods, (2) providing a reasonable basis for questionnaires according to past research results or expert opinions, (3) reducing biases that may result from group interaction, (4) ensuring the anonymity of the respondents in surveys, and (5) providing meaningful findings to participants [122,123]. Moreover, according to the responses collected from a limited number of experts (e.g., three mails, as mentioned by Brooks [124]), reaching consensus is easy. Typical applications of the modified Delphi method include summarizing experts’ opinions toward factors to select an enterprise resource planning (ERP) system for small and medium-sized enterprise under uncertainty [125], summarizing experts’ opinions toward factors influencing adoption of Internet of Things (IoT)-based wearable fitness trackers by Kao et al. [126], etc.

### 3.2. The DEMATEL Method

The DEMATEL method was proposed by Gabus and Fontela [111] of the Geneva Research Centre at the Battelle Memorial Institute to convert a complicated world problem into a clearly demonstrated influential relationship amongst the root causes of the problem. The major concept of this technique is to define a set of influential relationships based on a network structure. DEMATEL aims to use matrices to construct direct and indirect influential relationships and assist in identifying the influential strength among the aspects/criteria being considered. Unlike the traditional statistical methods (e.g., correlation analysis or structural equation modelling), which require an especially large number of samples to derive the causal relationships among variables based on correlation variables, the DEMATEL method needs only the opinions provided by a limited number of respondents [127] to derive acceptable influential relationships [128]. Please refer to Appendix A of [129] for these detailed procedures.

### 3.3. The DANP Technique

The DANP method was proposed by Professor Gwo-Hshung Tzeng [112,113]. The major purpose of DANP is to transform the total influence matrix derived by DEMATEL to the unweighted supermatrix and then derive the influence weight associated with each aspect and criterion accordingly. The major goal of introducing the DEMATEL technique was to utilize the graph theory of discrete mathematics to investigate complicated practical problems. The qualitative and mutually influenced aspects of decision-making problems can be resolved. DANP can reflect the essence of a decision problem by preventing the loss of any information due to the trimming of any influential relationships caused by defining a threshold value and the simplifying of the investigation process by reducing the survey time required for conventional ANP processes. Please refer to Appendix C of [129] for these detailed procedures.

### 3.4. VIKOR

VIKOR is a compromise-ranking technique that can be integrated with other MCDM methods [115] to resolve a complex decision problem. Grounded on the thought of compromise solution proposed by Yu [130] as well as Zeleny and Cochrane [131], the optimal alternative can be derived using the VIKOR method. The compromise solution is a feasible one that is the nearest to the aspired solution. The word “compromise” means that the solution can be derived from a consensus reached by mutual concessions [132]. For a decision problem with conflicting criteria, a compromise solution can support the decision-makers to develop a final decision [132]. Unlike the TOPSIS method proposed by Hwang and Yoon [133,134], the traditional method for deriving the compromise-ranking solution with the shortest distance to the ideal solution and the farthest from the worst solution, VIKOR considers the relative importance of these two distances and develops a rational compromise solution based on the maximum group utility of the majority (represented by min *S*) and a minimum of the maximum individual regret of the opponent (represented by min *Q*).

Based on the methods discussed thus far, this paper employed the DANP procedures to derive the weights associated with the aspects and criteria by considering the dependence and feedback among them. It then employed the modified VIKOR method to derive a compromise solution as the most suitable heterogeneous R&D alliances for an IVD company in a rapidly catching-up economy. For detailed VIKOR processes, please refer to Appendix D of the first author’s earlier work [129].

## 4. Results

The IVD industry is characterized as having knowledge workers, a long R&D cycle, huge capital expenditures, and high uncertainties. However, most IVD firms in late-coming economies are small and medium in size and have extremely limited resources. Determining how these IVD SMEs can leverage external resources to optimize the R&D performance has become a critical issue for the late-coming small and medium IVD firms. R&D alliances in general, and heterogeneous R&D alliances in particular, are critical for releasing the resource limitation of late-coming SMEs and reaching the metaoptimum of the R&D performances. In order to derive the evaluation criteria for selecting the strategies of the heterogeneous alliance of small and medium IVD firms, this section presents an empirical study based on the modified DANP followed by the modified VIKOR. Based on the aspects and criteria being summarized in Table 1, the modified Delphi method is used to filter out the more important criteria, as summarized in Section 4.1. As any aspects and criteria being derived by the modified Delphi may influence one another, the major relevance is identified using DEMATEL in Section 1. The DANP is employed to derive the influential relationships between each criterion in the same subsection. Finally, in Section 4.3, the modified VIKOR method is applied to evaluate and select the most suitable heterogeneous R&D alliance strategies. Based upon the VIKOR results, the heterogeneous R&D alliance strategies are derived.

### 4.1. Criterion Definition by Modified Delphi Method

In order to investigate the related factors influencing the selection of the heterogeneous R&D alliance strategies, the modified Delphi method was applied to summarize the feasibility of the criteria being proposed in Table 1 based on the opinions being provided by nine Taiwanese experts (refer Table 2) with at least 17 years of work experiences. According to the definition of the modified Delphi method (see Section 3.1), the 75% ratio was defined as the minimum percentage of agreement on any particular criterion.

Table 3 indicates the number of experts agreeing with the related factors influencing the selection of the heterogeneous R&D alliance strategies in the biotechnology industry. All ratios exceeded 75%. Thus, all of these requirements were considered suitable for predicting the IVD firms’ acceptances of the heterogeneous R&D alliance strategies.

### 4.2. Decision Problem Structuring

Based on the nine experts’ opinions, the causal relationship and the weights versus criteria were derived as follows. The criteria of this study are shown in Table 1. Here, the initial direct influence matrix D (refer Table 4) was obtained based on the experts’ opinions; it revealed the original interrelationships among these 19 criteria based on each expert’s viewpoint. The experts were asked to indicate the direct effect that they believed factor i would have on factor j, as indicated by $d_{ij}$.

Briefly, the significant confidence test being proposed by Lu et al. [119] was introduced to verify whether the experts’ opinions are consistent based on the initial influence matrix in Table 4. At first, the hypotheses are formulated as $H_{a0}^{}$: the experts’ opinions on the influence levels from any one criterion on the other criteria are consistent. The alternative hypothesis can be formulated as $H_{a1}^{}$: the experts’ opinions on the influence levels from any one criterion on the other criteria are inconsistent. Then, the level of significance is defined at α < 0.05. After that, the value of α can be derived following Lu et al. [119], where $\alpha =\frac{1}{n\times (n-1)}\sum_{i=1}^{n}\sum_{j=1}^{n}\frac{\left|d_{ij}^{p}-d_{ij}^{p-1}\right|}{d_{ij}^{p}}\times 100\%=1.456\%,$ which is smaller than 5%. That is, the significant confidence is 98.544%. Here, p=9 denotes the number of experts, $d_{ij}^{p}$ is the average influence of i criterion on j, and n denotes the number of criteria. Thus, $H_{a0}^{}$ is significant.

**Figure A1:**
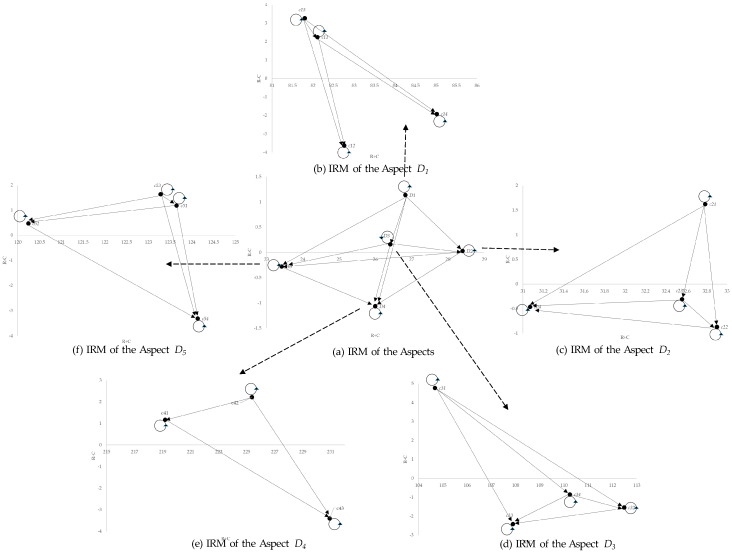
Causal relationship structure of total dimensions influences the heterogeneous R&D alliance strategies.

Furthermore, the causal relationship was structured based on the total relationship matrix T (refer to Table 5). The influential relationship was defined based on the threshold value. The threshold value of dimensions was 2.253. The causal relationships derived are shown in Figure A1 in Appendix A. The $\left(r_{i}+c_{i}\right)$ and $\left(r_{i}-c_{i}\right)$ are demonstrated in Table 6. The implication of $\left(r_{i}+c_{j}\right)$ represents the strength of the influence, both dispatching and receiving, while $\left(r_{i}+c_{j}\right)$ is the degree of the central role that factor *i* plays in the problem.

If $\left(r_{i}-c_{j}\right)$ is positive, the factor is primarily dispatching influence upon the other factors; if $\left(r_{i}-c_{j}\right)$ is negative, then the factor is primarily receiving influence from other factors. The three key factors influencing other factors are “obtain complementary competences,” “obtain technology and skills,” and “improve the level of innovation”; these are the most important factors for accepting heterogeneous R&D alliances in the IVD industry.

Among the 19 criteria, based on the influence degrees shown in Table 6, “obtain financial capital” (r + c = 19.768) is the most influential criterion, followed by “obtain the technology and skill” (r + c = 19.487) and “obtain the knowledge workers” (*r* + *c* = 19.468). In Figure A1, the transaction cost ($D_{1}$; minimize the costs of governing activities) is the most influential aspect, whereas resource dependency ($D_{4}$) is the aspect influenced by all other aspects. Based on the influence degrees demonstrated in Table 6, transaction costs influence resources ($D_{2}$; maximize the value of the company’s competences and capabilities), strategic behaviors ($D_{3}$; targeting specific market), and the learning of organization ($D_{5}$; accumulate the knowledge regarding technologies and know-how required). Resource dependency ($D_{4}$) is influenced by all other aspects. The rationality is discussed further in Section 5.

In the first transaction cost aspect, the criteria’s causal relationship can be structured based on the total relationship matrix corresponding to $D_{1}$. The causal relationships derived are shown in Figure A1b. The $\left(r_{i}+c_{i}\right)$ and $\left(r_{i}-c_{i}\right)$ are shown in Table 6. According to Figure A1b, $c_{13}$ “reduce transaction costs” influences “improve frequency” and “share uncertainly risks” through “asset specificity.” In the second resource-based aspect ($D_{2}$), according to the IRM demonstrated in Figure A1c, “obtain financial capital” ($c_{21}$) directly influences “obtain complementary competences” ($c_{22}$) and “obtain the technology and skills” ($c_{24}$). Furthermore, “obtain knowledge workers” ($c_{23}$) mediates the influential relationship. According to Figure A1d, in the third strategic behavior aspect ($D_{3}$), “improve the reputation” ($c_{31}$) influences “gain the new cooperation” ($c_{32}$) and “improve competitive relationship” ($c_{33}$). “Achieve the special goal” ($c_{34}$) mediates the influential relationship. In the fourth resource dependency ($D_{4}$) aspect, “achieve subsidy qualification” ($c_{42}$) directly influences “improve local competitive advantages” ($c_{43}$). “Overcome governmental barriers” ($c_{41}$) also mediates the influential relationship. In the fifth organizational learning dimension ($D_{5}$), the IRM can be structured. The causal relationships derived are demonstrated in Figure A1e. In the subgraph, “promote the learning process” ($c_{53}$) influences the “cultivation of talents” ($c_{54}$). “Improve the level of innovation” ($c_{51}$) and “gain experiences” ($c_{52}$) mediate the influential relationships.

After deriving the IRM, the weights associated with those criteria were derived using DANP. First, the total-relation matrix T was taken as the input to derive the influence weights versus criteria. The criteria weights were normalized using the influence matrix $T_{D}$ (this process was introduced in Section 3.3). Based on the normalized total-influence matrix $T_{D}$, the unweighted supermatrix W (Table A1 in Appendix B) was derived using Equation (A13) from [129] by the first author. These total influence values were used to derive the weighted supermatrix. Finally, by raising the power of the unweighted supermatrix to infinity using Equation (A15) from [129] by the first author, the stable supermatrix (Table A2 in Appendix B) and the influence weights versus criteria were derived.

According to the weights shown in Table 6, the resource was ranked as the most important weight, while the organizational learning was ranked as the least important one. From the viewpoint of weights being associated with the criteria, improve the local competitive advantage ($c_{43}$), overcome governmental barriers ($c_{42}$), and overcome governmental barriers ($c_{41}$) were ranked as the top criteria. Of the criteria belonging to the transaction cost ($D_{1}$) aspect, the organization learning aspect ($D_{4}$) and improving the reputation ($c_{31}$) were ranked as the least important ones.

### 4.3. Rank the Types of R&D Alliances by VIKOR

To enhance the adoption of heterogeneous R&D alliance strategies of the IVD industry, adoption strategies can be evaluated based on the criteria derived. Furthermore, the weights derived using DANP were introduced in Section 4.2. The literature identifies well-known R&D heterogeneous alliance types as: (1) equity-based: joint ventures, (2) nonequity-based: technology and patent licensing contracts, (3) nonequity-based: technology and patent sharing, and (4) nonequity-based: joint R&D projects. The types of the heterogeneous R&D alliance strategies used the modified Delphi method to filter out the suitable strategies. Nine IVD industry experts with extensive experience joined this study and summarized the opinions listed in Table 3. The average criterion performance scores versus each strategy are shown in Table A3 of Appendix C. The range of $f_{ik}$ is defined from 1 to 5, i.e., the aspiration level is 5 and the worst level is 1. So, $f_{i}^{*}=5$ and $f_{i}^{-}=1$.

By introducing the relative weight versus each criterion, the rank of $S_{k}\;(L_{p}=1)$ and $Q_{k}\;(L_{p}=\infty )$ was derived. $R_{k}$ was also derived by setting *v* as 0.5. Thus, the preferences of the heterogeneous R&D alliance adoption strategies could be evaluated and ranked, and the results are shown in Table A3.

The internal consistencies of the performance scores being graded by the experts were tested using the Cronbach’s alpha as follows. The null-hypothesis for the performance scores being associated with each strategy is defined as no difference between the scores being graded by the experts. The alternative hypothesis assumes the performances being graded by the experts are different. The hypotheses of the tested question for the performance scores of the four strategies can be formulated as follows. $H_{b0}^{}$: the performance scores for the “joint venture” strategy by the experts are consistent. The alternative hypothesis can be formulated as: $H_{b1}^{}$: the performance scores for the “joint venture” strategy by the experts are inconsistent. $H_{c0}^{}$: the performance scores for the “technology and patent’s licensing contracts” strategy by the experts are consistent. $H_{c1}^{}$: the performance scores for the “technology and patent’s licensing contracts” strategy by the experts are inconsistent. $H_{d0}^{}$: the performance scores for the “technology and patent’s sharing” strategy by the experts are consistent. $H_{d1}^{}$: the performance scores for the “technology and patent’s sharing” strategy by the experts are inconsistent. Finally, for the joint R&D strategy, the null hypothesis can be formulated as $H_{e0}^{}$: the performance scores on the “joint R&D” strategy by the experts are consistent, while the alternative hypothesis can be formulated as $H_{e1}^{}$: the performance scores on the “joint R&D” strategy by the experts are inconsistent. The Cronbach’s alpha values being associated with the four hypotheses are 0.869, 0.754, 0.890, and 0.847, respectively. Thus, $H_{b0}^{}$_,_ $H_{c0}^{}$_,_ $H_{d0}^{}$ and $H_{e0}^{}$ are significant. According to the VIKOR results, the joint venture was ranked first, followed by joint R&D, technology and patent sharing contracts, and technology and patent licensing contracts.

## 5. Discussion

This research sought to establish an analytic framework for identifying the key success factors used to define the heterogeneous R&D strategic alliance strategies for SMEs in the IVD industry. Meanwhile, adoption strategies were derived with VIKOR based on the influence weights versus criteria using DANP based on experts’ opinions. The most suitable heterogeneous R&D alliance strategies were then selected for the late-coming small and medium-sized IVDs. In this section, analytic results, comparisons of the analytic results with past works, and strategic implications will be discussed.

### 5.1. Influential Relationships between Aspects

Previous studies have integrated well-known theoretic frameworks related to heterogeneous R&D alliances, including transaction cost, resource-based viewpoint, strategic behavior, resource dependence, and organizational learning theories. The modified Delphi method was used to filter out the related criteria influencing the acceptance of strategies for heterogeneous R&D alliances (see Table 3). The influential relationships derived and demonstrated are consistent with past works. According to the IRM shown in Figure A1, the transaction costs influence resource dependency. Meanwhile, the resource-based viewpoint, strategic behavior, and organizational learning mediate the influential relationship.

At first, from the aspect of transaction costs ($D_{1}$), transaction costs’ influence on resources is consistent with earlier works. According to Fahy and Smithee [135], a sustainable competitive advantage stemming from resource heterogeneity can be anticipated to result in superior performance or rent. However, to ensure that the level of such returns is not overstated, considering the cost of resource deployment is essential. Suematsu [10] argued that a transaction cost is the cost generated when searching for a partner, gathering information, negotiating and reaching a decision, exchanging, as well as engaging in expost exchanges. Transactions are more effective while extra resources are consumed in each of the components. Because resources are rare and limited, transaction cost management is not only a cost reduction problem but it is also an issue of resource allocation [10]. Transactions, in addition to purchase and sales activities between and within the firm, include all communication and interactions within the firm [10]. The transaction cost seems to influence the availability of resources.

The phenomenon is especially significant for the IVD industry. According to Peeling and McNerney [1], whose information was published by the WHO, high-quality diagnostic technologies can be easily obtained for infectious diseases in advanced countries. However, these technologies are hard or impossible for most developing countries or economies to get where disease problems are serious. The limited investments for R&D of diagnostics, a state complicated by a deficiency of know-how to predict the market scale and customer needs, mean product specification and pricing are seriously needed due to the shortage of clear marketing channels and mechanisms for commercializing products developed, the shortage of transparent and clear standards in regulatory approval mechanisms, the long approval procedures of regulations, the shortage of regional coordination, and the limited scale of domestic markets, which cannot support low-cost manufacturing. These are fundamental problems faced by the IVD industry of rapidly catching-up or developing economies. The shortage of transaction costs limits the availability of resources and, thus, the successful commercialization of IVDs.

Transaction cost ($D_{1}$) also influences resource dependency ($D_{4}$) through the resource-based view ($D_{2}$), strategic behavior ($D_{3}$), and organizational learning ($D_{5}$). For the influences from transaction costs to the resource-based view, as the authors previously discussed in the second paragraph of this subsection, transaction cost management is not only a matter of cost reduction but it is also a matter of resource allocation [10]. Transactions, other than purchase and sales activities between and within the firm, include all communication and interactions within the firm [10]. Thus, transaction cost influences the availability of resources.

For the influences of transaction costs on strategic behavior, according to Jones and Hill [136], transaction costs include the negotiation, monitoring, and enforcement costs required in an exchange between two parties. These costs stem from the transaction difficulties that may exist in the exchange procedure [13,137]. By extending the work of [13,137], Dundas and Richardson [138], Kay [139,140], Teece [141], as well as Jones and Hill [136], it can be defined that the selection process of a strategy structure is a function of both factors: (1) the economic benefits gained by the decrease in transaction costs when a strategy is defined to internalize transactions within the firm and (2) the bureaucratic costs associated with managing the resultant intrafirm exchange. Transaction costs ($D_{1}$) influence strategy and, thus, strategic behavior ($D_{3}$).

Regarding the influential relationships of transaction cost ($D_{1}$) on organizational learning ($D_{5}$), according to the analytic results by Verwaal, Verdu and Recter [142], organizational learning is a vital complement to transaction cost efficiency, especially from the aspect of forming strategic outsourcing relationships. Transaction cost economics has had an enormous impact on the theories of economic exchange and the development of organizational processes and practices [143], in general.

For the influences from the resource-based view ($D_{2}$) to strategic behavior ($D_{3}$), according to the definition by Grant [144], a strategy is the match between the in-house resources and skills of a firm and the opportunities/chances and risks created by the external environment. The in-house resources can be further classified into seven categories: financial, physical, legal, human, organizational, informational, and relational. According to Chandler and Hanks [145], particular resource-based competences are associated with a firm’s specified competitive strategies [145]. An alliance can be a relational resource when cooperation is produced among partners [146]. According to Smith and Graetz [147], resource theory begins with the assumption that knowledge about resources, including their abundance, scarcity, and ownership, contributes to effective strategic decision-making and allows an organization to build a solid, sustainable base [148,149]. Thus, the resource-based view ($D_{2}$) affects strategic behaviors ($D_{3}$).

In terms of the influences of the resource-based view ($D_{2}$) on organizational learning ($D_{5}$), Doz and Hamel [150], as well as Das and Teng [78], argued that strategic alliances are specifically created to maximize firm values by integrating and taking advantage of the valuable resources of partner(s). Valuable resources ($D_{2}$) are usually rare, cannot be imitated easily, and cannot be moved easily; thus, gathering of these resources ($D_{2}$) is strategically necessary for a firm. Engaging in behavioral routines of acquiring, analyzing, and disseminating experiential alliance learning through the organization ($D_{5}$) may allow one partner to obtain insufficient competences when a strategic alliance is formed. These late-adopted competencies may result in performance improvement [151]. Based on the abovementioned works, the resource-based view ($D_{2}$) affects organizational learning ($D_{5}$).

The influences of strategic behaviors ($D_{3}$) on the resource-based view ($D_{2}$), organizational learning ($D_{5}$), and, consequently, resource dependency ($D_{4}$) are also consistent with past works. In terms of the influences of strategic behaviors ($D_{3}$) and, thus, the resource-based view ($D_{2}$), corporate strategy is defined as the long-lasting direction and scope of a firm, which is beneficial for the firm through the formation and application of resources within a varying environment to achieve its goals [152]. Against this background, top management is expected to have a total perspective and, as such, be involved in organizational strategies to acquire resources that assist in furthering the accomplishment of these objectives [153,154]. Burgelman [155] argued that innovations in firms are the outcomes of the induced strategic behavior ($D_{3}$) and autonomous strategic behavior ($D_{3}$), both of which are correlated with the firms’ strategic concepts. The induced strategic behavior ($D_{3}$) is regarded as the formal pathway for innovation, whereas autonomous strategic behavior ($D_{3}$) is the pathway where entrepreneurial participants, at the product and market level, think of novel business opportunities, participate in the efforts of the project champion for mobilizing resources ($D_{2}$ and $D_{4}$) of the firm for these new opportunities, and perform strategic forcing efforts to generate momentum for further development ([156], p. 156).

For influences of strategic behaviors ($D_{3}$) on organizational learning ($D_{5}$), according to Fiol and Lyles [157], the strategic position of an organization partly decides its learning capacity. Strategies are defined based on the goals and objectives as well as the scope of actions available for executing the strategy [157]. Therefore, strategy impacts organizational learning ($D_{5}$) by limiting decision-making and the context for the perception and interpretation of the environment [158,159,160]. Likewise, the strategic choices perceived are a function of the learning capacity ($D_{5}$) of the organization [155]. The strategic position also generates momentum for organizational learning ($D_{5}$) [157].

Regarding the influences of strategic behaviors ($D_{3}$) on resource dependency ($D_{4}$), according to Bridoux [161], the firm could select to build the resource internally when the resource is unavailable or when the acquisition of the external resource is more expensive than resource building. Mathews [162], who regarded external resource acquisition ($D_{4}$) as a dominant strategic alternative ($D_{3}$), compares the possible competitive advantages and disadvantages of searching through various outer sources ($D_{4}$) with those of establishing internal resources ($D_{3}$). In his view, the features of the resource build-up process (uneconomical time compression, asset quality efficiency, asset stock interlinkages, asset erosion prevention, and causal ambiguity) should facilitate the internal development of resources ($D_{2}$) [163] and, in some cases, may become a competitive disadvantage. In biotechnology and pharmaceuticals, extensive markets for technologies and know-how have developed over the past decade, making external resource acquisition ($D_{4}$) a dominant strategic alternative ($D_{3}$) [161,162].

In terms of the influences of internal resources ($D_{2}$) on the acquisition of external resources ($D_{4}$), according to Tehseen and Ramayah [164], SMEs have rare resources ($D_{2}$) of finance, skills, technology, and knowledge; hence, the sustainable business success of SMEs is greatly dependent on numerous other factors that include the supplier’s competences and the integration of the customer’s competences [164]. Therefore, whether a firm intends to acquire external resources ($D_{4}$) depends on the internal resources ($D_{2}$) and competencies owned by the specific firm, so those with internal resources ($D_{2}$) can influence the acquisition of external resources ($D_{4}$).

### 5.2. Most Influential and Important Criteria

This subsection further discusses the most influential and important criteria based on the degrees of influence demonstrated in Figure A1 and Table 6. In the first transaction cost ($D_{1}$) aspect, the reduction of transaction cost ($c_{13}$) and asset specificity ($c_{11}$) are the most influential factors. These results are consistent with Williamson’s view of transaction cost economics [14,165,166] based on the interaction among the three important dimensions, namely, uncertainty, asset specificity ($c_{11}$), and transaction frequency ($c_{14}$), of a transaction. According to Teoet al. [86] empirical study results, transaction cost and asset specificity ($c_{11}$) are positively correlated with product uncertainty and behavior uncertainty. Thus, when the transaction cost is reduced, both asset specificity and uncertainties associated with product and behavior decrease. These results are consistent with previous research. However, the work also proposed a new insight regarding theoretical development. Except for the impacts of uncertainty, asset specificity, and transaction frequency, the transaction cost will influence these three factors.

In the second internal resource ($D_{2}$) aspect, “obtain financial capital” ($c_{21}$) is the most influential criterion. However, the weight associated with the criteria is ranked lower (Rank = 17). Initially, the financial capital can influence the acquisition of knowledge workers ($c_{23}$), technology and skills ($c_{22}$), and complementary competences ($c_{24}$). The knowledge workers ($c_{23}$) then mediate the influential relationships, which are straightforward and consistent with past findings. According to Pfeffer and Leblebici [167], a firm’s financial structure can influence the recruitment of executives; the poorer the financial condition, the greater the executive turnover. Stahl, Björkman, Farndale, Morris, Paauwe, Stiles, Trevor and Wright [168] subsequently argued that retaining human resources requires a multifaceted approach. Competitive compensation is certainly necessary for attracting and retaining top human resources, but firms are also increasingly aware that financial incentives are only one of the key success factors. According to Chyi Lee and Yang [169], a knowledge worker has the ability, knowledge, and skills in an organization (e.g., engineer and accountant). Thus, the recruitment of knowledge workers can help organizations acquire knowledge as well as technology, skills ($c_{22}$), and complementary competences ($c_{24}$).

In the third strategic behavior ($D_{3}$) aspect, “improve the reputation” ($c_{31}$) is the most influential criteria. According to Fu, Hauert, Nowak and Wang [170], persons with decent reputations ($c_{31}$) are more likely to appeal to new partnerships ($c_{32},\;c_{34}$), while those with low reputations can lose current partnerships. Reputation represents the basic quantity of the company’s products and services. Consumers may be willing to pay a premium for the products of reputable companies, especially in uncertain markets [96,97]. The concept of reputation as “perceived quality” [171] refers to the firm’s capabilities to create values that are positively appraised by stakeholders [172]. A firm with a good reputation could also retain a cost advantage because, with other conditions remaining the same, employees prefer working for firms with a strong reputation and, thus, must work harder or for worse payment [97]. At the same time, as suppliers are less concerned with risks associated with contracts while dealing with firms with better reputations, decent reputations should also result in lower costs for contracting and monitoring [97]. According to Roberts and Dowling [97], reputation plays a dominant role when uncertainties about the quality of a firm’s products/services exist. The same uncertainty makes it tough for competitors to demonstrate quality rapidly, which would offset the signaling benefits accompanying a decent reputation [97]. Thus, an increase in a firm’s reputation can alter the competitive situation ($c_{33}$).

Furthermore, Granovetter [98], and Hill [99] argued that reputation is a dominant factor of a successful alliance. Reputation is valuable to a possible partner because the value of both the firm and the asset that can be obtained in an alliance can be evaluated accordingly. In addition, a positive reputation indicates that a partner is truthful and reduces the perceived possibility of a wrong partner. Consequently, an improved firm reputation ($c_{31}$) can lead to new strategic alliances ($c_{34}$) and form new partnerships ($c_{32}$). Finally, the competitive situation ($c_{33}$) can be changed.

In the fourth external resource aspect ($D_{4}$), “achieve subsidy qualification” ($c_{42}$) is the most influential criterion. In many countries, governments grant different capital subsidies to the business sector in order to promote growth [104]. To be eligible for a subsidy, an individual or couple must have countable resources. Based on the subsidies being granted, competitive advantages ($c_{43}$) can be achieved and governmental barriers ($c_{41}$) can be easily overcome.

In the fifth organizational learning aspect ($D_{5}$), “promote the learning process” ($c_{53}$) and “improve the level of innovation” ($c_{51}$) are the most influential criteria. The IVD industry needs a wide variety of technological domain-related know-how. The primary motivation for a company to join an alliance is to learn (e.g., transfer and absorb the partner’s knowledge to discover new knowledge) or acquire, examine, and retrieve the partner’s knowledge assets to take advantage of complementarity [173].

### 5.3. Strategic Implications Based on Compromised Solutions by VIKOR

For the heterogeneous R&D alliance adoption strategy of IVD industry definitions by VIKOR, the multiple criteria decision analysis (MCDA) is appropriate for solving decision-making problems involving various aspects. The modified VIKOR method is one of the easiest and most reasonable approaches to apply among the MCDA’s existing ranking methods. Table A3 in Appendix C presents the scores based on the weights derived from DANP.

In this research, *v* = 0.5 is selected to seek maximum group utility of the majority and the minimum individual regret of the “opponent.” Because $R_{k}$ is the value of desire in a small index ($R_{k}$ as small as possible) and the ranges of $R_{k}$ are set from 0 to 1, this research transforms it into the value of desire in a big index ($1-R_{k}$ as big as possible). In particular, $1-R_{k}$ is the VSI value (see Table A3). When *v* = 0.5, the types of heterogeneous R&D alliance strategies of the IVD industry could be ranked as follows: joint ventures > joint R&D projects > technology and patent sharing contracts > technology and patent licensing contracts. In this study, nonequity-based alliances are a better heterogeneous R&D alliance adoption strategy based on the IVD industry definitions in VIKOR than equity-based alliances, especially the joint R&D project strategy. The strategic implications based on compromise solutions derived by VIKOR will be discussed next. Based on the VIKOR results, strategic proposals will be discussed along with support from previous literature.

The joint venture was selected as the most suitable strategy due to the better scores in “reduction of transaction costs” ($c_{13}$), “obtain complementary competences” ($c_{24}$), “strategic behavior” ($D_{3}$), “resource dependency” ($D_{4}$), and “enhance organizational learning” ($c_{53}$) (see Table A3).

Hennart [174] recommended a hybrid model between the market (fair trading agreement) and the hierarchy (wholly owned subsidiary) to reduce transaction costs ($c_{13}$), facilitate the pooling of complementary resources ($c_{24}$), and reduce risk. Kogut [27] argued that high uncertainties inspire the establishment of joint ventures while a firm’s performance is strongly influenced. Consequently, alliances are shaped as a protection mechanism to cope with strategic uncertainties. Kogut argued that joint ventures are used to transfer the knowledge embedded in an organization (i.e., organizational learning ($c_{53}$)) that cannot be easily transferred [27].

According to Dollinger, Golden and Saxton [175], the positive reputation of the target firm increases the tendency of decision-makers to participate in joint ventures. Furthermore, based on the discussion in Section 5.1, the strategic behavior ($D_{3}$) to improve reputation ($c_{31}$) influences the competitive situation ($c_{33}$) through the development of new strategic partnerships ($c_{34}$) and new cooperation efforts ($c_{32}$). Thus, in terms of strategic behaviors ($D_{3}$), in general, and the improvement of reputation ($c_{31}$), in particular, selecting the joint venture strategy is very straightforward.

Finally, from the aspect of resource dependency ($D_{4}$), joint ventures are shaped by partner firms to pursue a wide variety of strategic objectives and jointly defined goals not achievable by acting separately [176,177]. The objectives can include: (1) reducing costs (e.g., achieving economies of scale), (2) accessing markets, (3) accessing know-how, and (4) reducing risks (e.g., sharing investments) [178,179,180,181]. Nowadays, technology evolves rapidly while the life cycle of a new technology is extremely short. Investing in a novel technology in general, and an IVD technology in particular, is beyond the scope of a firm in a developing economy, even with a massive amount of government subsidies [182]. Our empirical study results from the IVD industry are also consistent with the earlier observation of Teece [183], who found that collaboration in IVD mainly involves marketing only, supply, or technology transfer. Joint ventures between contractors in developing countries have been recognized as a feasible mechanism for technology transfer and, thus, a feasible method for improving insufficient skills [180,184,185,186,187,188]. In Taiwan’s high-technology industries (e.g., pharmaceutical and IVD industries), a laissez-faire approach has been widely adopted while the techniques of mass production are mainly based on licensing [189].

Finally, in the transfer of IVD technology to developing countries, the advantage of local manufacturing over imports is the reduction of regulatory barriers ($c_{41}$), foreign currency expenditures, and access and transport costs, thereby increasing local competitive advantage ($c_{43}$). Although most firms believe that the lack of regulations is damaging the industry, some firms regard the opportunity to sell their tests without restriction as opportunities.

## 6. Conclusions

The IVD industry is considered one of the most important industries of the 21st century and offers tremendous value to humankind. In addition to offering economic benefits, it also contributes to the discovery and development of medical products, which can extend the lifespan of human beings and improve health and quality of life. The IVD industry is highly technological and is characterized as having knowledge workers, a long R&D cycle, huge capital expenditures, and high levels of uncertainty. However, most IVD firms in late-coming economies are small and medium-sized firms with very limited resources. This study explored how firms can leverage external resources to optimize their R&D performance. R&D alliances, in general, and heterogeneous alliances, in particular, are critical for eliminating the resource limitations of late-coming SMEs and reaching the metaoptimum of R&D performances. This research identified the key success factors for such heterogeneous alliances. Possible factors were identified from the existing literature and confirmed by experts using the modified Delphi method. DEMATEL was then introduced to structure the causal relationships between criteria. DANP was used to derive the weights versus the criteria. The performance scores versus the strategies were calculated accordingly using VIKOR. An empirical study based on major Taiwanese IVD firms’ evaluation and selection of heterogeneous R&D alliance strategies was used to demonstrate the feasibility of the proposed analytic framework.

The results of this study show that heterogeneous R&D alliances’ motivations are positively related to R&D performance, and the most important factor for accepting a heterogeneous R&D alliance strategy is organizational learning. Existing literature supports this study’s conclusion that heterogeneous R&D alliances would be a useful strategy to improve the R&D performance of late-coming SMEs in the IVD industry and increase their growth. According to the analytic results of this research, obtaining complementary competences is the top priority. Organizational learning is the most important construct for accepting heterogeneous R&D alliances, and joint R&D projects are a suitable type of heterogeneous R&D alliance for late-coming SMEs in the IVD industry. This study investigated only the IVD industry. The pharmaceutical, diagnosis, and biotech service sectors can be investigated further in the future. Finally, the analytic framework and the strategic proposals presented in this study can be applied to other industries in future research on heterogeneous R&D alliances.

### 6.1. Economic and Social Implications

The availability and affordability of IVD devices are especially important for developing and low-income countries. Preparation for emergencies, assurance of the efficiency of health systems, and health equity can be guaranteed [190]. However, those IVD technologies or devices which are widely available in advanced countries are usually unavailable or unaffordable for middle- to low-income economies. The costs of imported IVD devices are too high for the public sector. Meanwhile, IVD vendors usually have problems understanding local markets of developing and less-developed economies, overcoming governmental barriers, and providing support to healthcare sectors, which are drastically underfunded in the least developed countries.

The proposed strategies for heterogeneous R&D alliances can effectively solve the unavailability and unaffordability problems of IVD devices. Thanks to lower R&D and production costs of devices provided by firms located in developing economies (e.g., China and India), low-cost substitute IVDs can be made available. Furthermore, IVD firms located in developing countries usually have good relationships with state and local bureaucracies and administrations as well as a better understanding of their own national and local health systems. These firms can cross governmental and policy barriers more easily. On-time and local supports are also more easily available, as they are provided by firms located in the same geographical region. Preparation for emergencies, assurance of the efficiency of health systems, and health equity can thus be guaranteed. More work opportunities, economic growth, and improved social welfare can, therefore, be made available.

### 6.2. Managerial Implications

While developing a new product in general, and a novel IVD device, in particular, collaboration is always a very important strategic option for high-technology firms. The major advantages include quicker acquisition of needed skills or resources, reducing asset commitment and increasing flexibility, learning from partners, and sharing costs and risks [191]. According to [192], the findings suggest that firms with a greater number of heterogeneous types of partners have greater benefits in terms of innovative performance, particularly when they draw deeply from a limited number of preferred partnership types. Based on the analytic results, heterogeneous R&D alliances are especially suitable for IVD firms in rapidly catching-up economies. In comparison with tier-one IVD firms in developed countries, IVD firms in developing countries are always short of assets and resources required for developing novel IVD devices. Therefore, appropriate heterogeneous R&D alliance strategies are very important for the innovation performance of IVD firms in fast catch-up economies.

Several managerial implications of the proposed analytic framework and the analytic results can be derived. First, the proposed analytic framework has strong applicability for the high-technology industries of fast catch-up economies in general and IVD industries, in particular. Managers can adopt the proposed analytic framework to evaluate and select suitable strategies for R&D alliances. Meanwhile, the joint venture strategy can be adopted by small and medium-sized IVD firms located in developing economies. In an era in which IVD firms are facing rapid evolution of technology and cost pressures, heterogeneous R&D alliances based on joint ventures can effectively solve the firms’ problems in quickly entering fast-emerging technical domains, such as, A.I., robots, communications, etc. The selected strategies can be further enhanced from their current status to the aspired level identified by VIKOR based on the influence relationships being derived. Finally, the joint venture strategy is not a panacea, especially in a knowledge-based economy. It is rarely possible for a joint venture partner to possess all the assets required for innovation. This is also consistent with our research results. The gap between the current VIKOR score (see Table A3) associated with the joint venture strategy and the aspired level (5.0) still exists. Strategies like open innovation or technology transfer are still required to fill the gap.

### 6.3. Limitations and Future Research Possibilities

Despite its contributions, this paper has some limitations that can be seen as promising avenues for future research. The major limitations of this research are twofold: (1) the experts were limited to Taiwanese experts whose opinions may be controversial and (2) the IVD industry widely includes areas such as agriculture, food, pharmaceuticals, and diagnosis [193]. However, this study investigated only the pharmaceutical, diagnosis, and biotech service sectors. Future research could collect global experts’ opinions on this issue, and the analytic framework and strategic proposals proposed in this study can be applied to other industries in future research on heterogeneous R&D alliances.

## Figures and Tables

**Table 1:** Criteria descriptions and rationalities.

Construct	Criteria (Symbol)	Description and Rationality	Source
Transaction Cost (TC)	Asset specificity (*c*_11_)	According to Teo, Wang and Leong [86], transaction cost and asset specificity are positively correlated with product uncertainty and behavior uncertainty.	[13,14,15]
	Share uncertainly risks (*c*_12_)	Uncertainty refers to unanticipated changes in the environment in which the transaction is embedded [87]. According to Wever, Wognum, Trienekens and Omta [88], level of uncertainty influences transaction risks and thus, transaction cost.	[13,14,15]
	Reduction of transaction costs (*c*_13_)	One of the major gains generated for the customers in vertical partnerships include reduction of transaction costs [89]. Therefore, whether a heterogeneous R&D alliance strategy can reduce the transaction cost is a very important criterion.	[89]
	Improve frequency (c_14_)	In transaction cost economics, dimensions for describing transactions are (1) assets’ specificity, (2) uncertainty, and (3) frequency [13,14,15]. The frequency of transactions will influence the cost of transactions.	[13,14,15]
Resource-Based Perspective (RB)	Obtain the financial capital (*c*_21_)	According to Mowery, Oxley and Silverman [90], the access to financial resources is one of the major motives for joint venture from the aspect of market access and others,	[91,92]
	Obtain the technology and skill (*c*_22_)	Based on the same work by [90], capturing and absorbing tacit know-how, extracting skills, performing basic research, and high costs and risks of R&D are the major technology-based motives for joint ventures.	[24,25,26,91]
	Obtain the knowledge workers (*c*_23_)	The resource-based theory of the firm argues that a business enterprise is best viewed as a collection of sticky and difficult-to-imitate resources, which include knowledge of specific markets or user needs for handling the marketing and distribution of products [90]. However, such strategic knowledge is embedded in employees themselves [93]. Therefore, whether a heterogeneous R&D alliance can help access the knowledge workers, and thus, the knowledge being embedded in these workers is very critical.	[92,94]
	Obtain complementary competences (*c*_24_)	A firm can develop a valuable and rare resource base to achieve a position of superiority. One typical approach is the creation of a bundle of complementary resources, including physical assets, intangible assets, and competencies, that produce customer value [95]. Tyler and Caner [57] explained that R&D alliances are formed for a variety of reasons, such as to access technological resources and capabilities, share R&D risks and costs, and explore new technological domains. Therefore, obtaining complementary competences is selected as the criterion for evaluating the appropriate strategy for heterogeneous R&D alliances.	[91]
Strategic Behavior (SB)	Improve the reputation (*c*_31_)	Reputation represents the basic quantity of the company’s products and services. Consumers may be willing to pay a premium for the products of reputable companies, especially in uncertain markets [96,97]. Granovetter [98] and Hill [99] argued that reputation is a dominant factor of a successful alliance. Reputation is valuable to a possible partner because the value of both the firm and the asset that can be obtained in an alliance can be evaluated accordingly.	[28,96,97,98,99]
	Gain new cooperation(s) (*c*_32_)	According to strategic behavior theory, firms establish cooperative arrangements in order to maximize long-term profitability by enhancing their competitive position [28]. The strategic behavior theory proposed by Kogut [27] and Jarillo [29] argued that firms enter cooperative alliances due to long-standing strategic considerations, regardless of instant concerns regarding costs and benefits [30]. In other words, strategic behavior theory predicts that firms will engage in cooperative activities as a means of achieving overall strategic objectives regardless of their effect on specific transaction costs [28].	[27,28,29,30]
	Improve competitive relationship (*c*_33_)	A competitive relationship is defined as the relationship between any two firms operating in the same industry and offering similar products (including services) [100]. Working with competitors, partners still enjoy indirect benefits on innovation; this explains why firms, understanding the downside of competitive R&D collaborations, still cooperate with competitors [101]. Therefore, whether a R&D alliance strategy can improve competitive relationship should be considered.	[102]
	Achieve the special goal (*c*_34_)	Strategic behavior theory predicts that firms will engage in cooperative activities as a means of achieving overall strategic objectives regardless of their effect on specific transaction costs [28].	[28]
Resource Dependency (RD)	Overcome governmental barriers (*c*_41_)	Most countries have a legal framework and a nominated body to regulate IVDs [103]. In countries that do regulate, approval for IVDs is often costly, lengthy, and, on occasion, lacking in transparency, thus regulation of diagnostics is currently seen as a barrier to innovation and access [103]. IVD firms can cooperate with vendors, access the most advanced techniques and quality systems, and overcome governmental barriers.	[103]
	Achieve subsidy qualification (*c*_42_)	In many countries, governments grant different capital subsidies to the business sector in order to promote growth [104]. To be eligible for a subsidy, an individual or couple must have countable resources.	[104]
	Improve local competitive advantages (*c*_43_)	To realize local competitive advantages, and thus to localize products and practices, subsidiaries need to apply those shared resources to the context [105]. Therefore, the strategy which can improve local competitive advantages should be selected.	[105].
Organizational Learning (OL)	Improve the level of innovation (*c*_51_)	According to [106], organizational learning capability influences product innovation performance. The criterion is selected accordingly.	[106]
	Gain experience (*c*_52_)	Organization learning is the process by which an organization identifies action relationship, identifies and corrects errors, and gains experience [107]. Thus, “gain experience” is defined as a criterion for the organizational learning aspect.	[107]
	Promote the learning process (*c*_53_)	The effectiveness of the organizational learning process depends on the levels of learning and knowledge transformation, retention, and creation within the organization. The key factor of organizational learning is how to leverage and motivate the learning process and practice at broader levels in the organization. Thus, a process for fostering the active interpersonal learning practices at the broader organizational level is critical [108].	[108]
	Cultivation of talent (*c*_54_)	According to [109], human-resource management is a determining factor in organizational learning. Especially, strategic training positively influences organizational learning. Therefore, the cultivation of talent is defined as a criterion for evaluating the most suitable strategy from the aspect of organization learning.	[109]

**Table 2:** Experts’ background.

Education	Industry	Title	Experiences
1. Ph.D.	IVD	President	25
2. Ph.D.	Pharmaceutical	President	30
3. Ph.D.	Diagnostic Medical Devices	Executive Vice President	25
4. Master	IVD	Vice President	23
5. Master	IVD	Director	19
6. Master	IVD	Director	17
7. Master	Biotech Services	Director	18
8. Ph.D.	Pharmaceutical	Director	22
9. Bachelor	Biotech Services	Manager	21

*Note.* This study.

**Table 3:** Questionnaire collected of experts’ opinions.

NO.	TC				RB				SB				RD			OL				Strategies			
	c_11_	c_12_	c_13_	c_14_	c_21_	c_22_	c_23_	c_24_	c_31_	c_32_	c_33_	c_34_	c_41_	c_42_	c_43_	c_51_	c_52_	c_53_	c_54_	JV	TPLC	TPSC	JRD
1	A	A	A	A	A	A	A	A	A	A	A	A	A	A	A	A	A	A	A	A	A	A	A
2	A	A	A	A	A	A	A	A	A	A	D	A	A	A	A	A	A	A	A	A	A	A	A
3	D	A	D	A	D	A	A	A	A	A	D	A	A	A	A	A	D	A	A	A	A	A	A
4	A	A	A	A	A	A	A	A	A	A	A	A	A	D	A	A	A	D	A	A	A	A	A
5	A	A	A	A	A	A	A	A	A	A	A	A	A	A	A	A	A	A	A	A	A	A	A
6	A	A	A	A	A	A	A	A	A	A	A	A	A	A	A	A	A	A	A	A	A	A	A
7	A	A	A	A	A	A	A	A	A	A	A	A	A	A	A	A	A	A	D	A	A	A	A
8	A	A	A	A	A	A	A	A	A	A	A	A	A	A	A	A	A	A	A	A	A	A	A
9	A	A	A	A	A	A	A	A	A	A	A	A	A	A	A	A	A	A	A	A	A	A	A
Agree	8	9	8	9	8	9	9	9	9	9	2	9	9	8	9	9	8	8	8	9	9	9	9
Disagree	1	0	1	0	1	0	0	0	0	0	7	0	0	1	0	0	1	1	1	0	0	0	0
Agree%	89%	100%	89%	100%	89%	100%	100%	100%	100%	100%	78%	100%	100%	89%	100%	100%	89%	89%	89%	100%	100%	100%	100%
Disagree %	11%	0%	11%	0%	11%	0%	0%	0%	0%	0%	22%	0%	0%	11%	0%	0%	11%	11%	11%	0%	0%	0%	0%

Remark: A, agree; D, disagree; JV, “joint venture”; JRD, “joint R&D”; TPSC, “technology and patent sharing contracts”; TPLC, “technology and patent licensing contracts.”

**Table 4:** The initial influence matrix ***D***.

5.000	3.778	3.222	3.778	3.861	3.861	3.861	3.861	3.639	3.639	3.639	3.639	3.861	3.861	3.861	3.417	3.417	3.417	3.417
3.222	5.000	3.111	3.556	3.750	3.750	3.750	3.750	3.528	3.528	3.528	3.528	3.750	3.750	3.750	3.306	3.306	3.306	3.306
3.333	3.667	5.000	3.889	3.875	3.875	3.875	3.875	3.653	3.653	3.653	3.653	3.875	3.875	3.875	3.431	3.431	3.431	3.431
3.444	3.667	3.444	5.000	3.833	3.833	3.833	3.833	3.611	3.611	3.611	3.611	3.833	3.833	3.833	3.389	3.389	3.389	3.389
4.111	4.111	4.111	4.111	5.000	4.333	4.667	4.222	4.111	4.111	4.111	4.111	4.000	4.000	4.000	3.944	3.944	3.944	3.944
3.958	3.958	3.958	3.958	4.111	5.000	4.000	3.889	3.958	3.958	3.958	3.958	3.847	3.847	3.847	3.792	3.792	3.792	3.792
3.972	3.972	3.972	3.972	4.000	4.444	5.000	3.667	3.972	3.972	3.972	3.972	3.861	3.861	3.861	3.806	3.806	3.806	3.806
3.875	3.875	3.875	3.875	3.444	4.111	3.778	5.000	3.875	3.875	3.875	3.875	3.764	3.764	3.764	3.708	3.708	3.708	3.708
3.639	3.639	3.639	3.639	3.972	3.972	3.972	3.972	5.000	4.111	3.778	3.778	3.750	3.750	3.750	3.417	3.417	3.417	3.417
3.667	3.667	3.667	3.667	4.000	4.000	4.000	4.000	3.889	5.000	4.000	4.000	3.778	3.778	3.778	3.444	3.444	3.444	3.444
3.569	3.569	3.569	3.569	3.903	3.903	3.903	3.903	3.000	4.000	5.000	4.111	3.681	3.681	3.681	3.347	3.347	3.347	3.347
3.639	3.639	3.639	3.639	3.972	3.972	3.972	3.972	3.444	4.222	4.000	5.000	3.750	3.750	3.750	3.417	3.417	3.417	3.417
3.537	3.537	3.537	3.537	3.537	3.537	3.537	3.537	3.648	3.648	3.648	3.648	5.000	3.222	3.667	3.148	3.148	3.148	3.148
3.593	3.593	3.593	3.593	3.593	3.593	3.593	3.593	3.704	3.704	3.704	3.704	3.333	5.000	3.889	3.204	3.204	3.204	3.204
3.593	3.593	3.593	3.593	3.593	3.593	3.593	3.593	3.704	3.704	3.704	3.704	3.444	3.778	5.000	3.204	3.204	3.204	3.204
3.403	3.403	3.403	3.403	3.903	3.903	3.903	3.903	3.347	3.347	3.347	3.347	3.514	3.514	3.514	5.000	4.333	3.778	4.333
2.944	2.944	2.944	2.944	3.833	3.833	3.833	3.833	3.278	3.278	3.278	3.278	3.444	3.444	3.444	3.889	5.000	4.000	4.000
2.979	2.979	2.979	2.979	3.903	3.903	3.903	3.903	3.347	3.347	3.347	3.347	3.514	3.514	3.514	4.111	3.889	5.000	4.444
2.944	2.944	2.944	2.944	3.833	3.833	3.833	3.833	3.278	3.278	3.278	3.278	3.444	3.444	3.444	4.111	3.556	4.222	5.000

**Table 5:** The total influence matrix ***T***.

0.484	0.475	0.459	0.475	0.502	0.511	0.508	0.503	0.475	0.488	0.484	0.485	0.487	0.488	0.492	0.466	0.464	0.466	0.470
0.445	0.475	0.442	0.457	0.484	0.492	0.490	0.485	0.458	0.470	0.467	0.468	0.469	0.471	0.474	0.449	0.447	0.449	0.453
0.464	0.475	0.484	0.479	0.504	0.513	0.510	0.506	0.478	0.490	0.487	0.487	0.489	0.491	0.494	0.468	0.466	0.468	0.472
0.460	0.469	0.458	0.487	0.498	0.506	0.503	0.499	0.471	0.483	0.480	0.481	0.483	0.484	0.487	0.462	0.460	0.461	0.466
0.519	0.527	0.518	0.527	0.568	0.569	0.570	0.559	0.530	0.544	0.540	0.540	0.538	0.539	0.543	0.521	0.518	0.520	0.525
0.496	0.503	0.495	0.504	0.533	0.554	0.538	0.532	0.506	0.519	0.516	0.516	0.514	0.515	0.519	0.497	0.495	0.497	0.502
0.498	0.505	0.497	0.506	0.534	0.549	0.553	0.531	0.508	0.522	0.518	0.519	0.516	0.518	0.521	0.499	0.497	0.499	0.504
0.483	0.490	0.482	0.491	0.512	0.529	0.522	0.533	0.493	0.506	0.502	0.503	0.500	0.502	0.506	0.484	0.482	0.484	0.489
0.472	0.479	0.471	0.480	0.510	0.519	0.516	0.512	0.499	0.501	0.493	0.494	0.492	0.494	0.497	0.473	0.471	0.472	0.477
0.477	0.483	0.475	0.484	0.515	0.524	0.521	0.516	0.489	0.516	0.500	0.501	0.496	0.498	0.501	0.477	0.475	0.476	0.481
0.462	0.469	0.461	0.469	0.499	0.508	0.505	0.500	0.464	0.489	0.499	0.488	0.481	0.483	0.486	0.462	0.460	0.461	0.466
0.472	0.479	0.471	0.480	0.510	0.519	0.516	0.512	0.479	0.502	0.496	0.509	0.492	0.494	0.497	0.473	0.471	0.472	0.477
0.443	0.449	0.441	0.450	0.474	0.482	0.480	0.475	0.453	0.465	0.462	0.462	0.479	0.457	0.466	0.441	0.439	0.440	0.444
0.451	0.457	0.450	0.458	0.483	0.491	0.488	0.484	0.461	0.473	0.470	0.471	0.465	0.488	0.477	0.449	0.447	0.448	0.452
0.451	0.457	0.449	0.458	0.483	0.491	0.488	0.484	0.461	0.473	0.470	0.471	0.466	0.472	0.491	0.449	0.447	0.448	0.452
0.460	0.466	0.459	0.467	0.500	0.508	0.505	0.501	0.469	0.481	0.477	0.478	0.479	0.481	0.484	0.484	0.474	0.468	0.480
0.435	0.442	0.434	0.442	0.478	0.487	0.484	0.480	0.449	0.460	0.457	0.458	0.459	0.460	0.464	0.451	0.463	0.452	0.456
0.444	0.451	0.443	0.451	0.489	0.497	0.494	0.490	0.458	0.470	0.467	0.468	0.469	0.470	0.474	0.463	0.458	0.473	0.471
0.435	0.442	0.434	0.442	0.479	0.487	0.484	0.480	0.449	0.460	0.457	0.458	0.459	0.460	0.464	0.454	0.445	0.455	0.469

**Table 6:** Strength of the influence, relationship toward other dimensions/criteria, weight, and ranks.

Dimensions/ Criteria	*r*	*c*	*r + c*	*r − c*	Weight	Rank of Weight
**Dimensions**						
TC	13.981	12.841	26.823	1.140	0.196	4
RB	14.216	14.180	28.396	0.036	0.216	1
SB	13.287	13.125	26.412	0.163	0.200	3
RD	12.466	13.529	25.995	−1.064	0.206	2
OL	11.571	11.846	23.416	−0.275	0.181	5
**Criteria**						
*c*_11_	42.180	39.925	82.104	2.255	0.0486	14
*c*_12_	39.570	43.187	82.757	(3.617)	0.0494	12
*c*_13_	42.535	39.260	81.795	3.276	0.0484	15
*c*_14_	41.551	43.464	85.015	(1.914)	0.0494	11
*c*_21_	17.206	15.578	32.785	1.628	0.0536	7
*c*_22_	16.016	16.882	32.898	(0.867)	0.0547	4
*c*_23_	16.127	16.429	32.556	(0.302)	0.0543	5
*c*_24_	15.305	15.765	31.070	(0.460)	0.0538	6
*c*_31_	54.724	49.944	104.668	4.781	0.0492	13
*c*_32_	55.470	57.007	112.477	(1.537)	0.0506	8
*c*_33_	52.742	55.147	107.890	(2.405)	0.0502	10
*c*_34_	54.701	55.540	110.242	(0.839)	0.0503	9
*c*_41_	110.172	109.000	219.172	1.172	0.0684	3
*c*_42_	113.796	111.573	225.370	2.223	0.0686	2
*c*_43_	113.760	117.155	230.915	(3.395)	0.0692	1
*c*_51_	32.825	31.720	64.545	1.105	0.0452	17
*c*_52_	30.932	30.511	61.443	0.421	0.0450	19
*c*_53_	32.096	31.738	63.834	0.359	0.0451	18
*c*_54_	31.357	33.242	64.600	(1.885)	0.0456	16

*Note.* This study.

## Appendix B. The Unweighted and Weighted Supermatrices

**Table A1:** Unweighted supermatrix.

0.256	0.245	0.244	0.245	0.248	0.248	0.248	0.248	0.248	0.248	0.248	0.248	0.248	0.248	0.248	0.248	0.248	0.248	0.248
0.251	0.261	0.250	0.250	0.252	0.252	0.252	0.252	0.252	0.252	0.252	0.252	0.252	0.252	0.252	0.252	0.252	0.252	0.252
0.243	0.243	0.255	0.245	0.248	0.248	0.248	0.248	0.248	0.248	0.248	0.248	0.248	0.248	0.248	0.248	0.248	0.248	0.248
0.251	0.251	0.252	0.260	0.252	0.252	0.252	0.252	0.252	0.252	0.252	0.252	0.252	0.252	0.252	0.252	0.252	0.252	0.252
0.248	0.248	0.248	0.248	0.251	0.247	0.246	0.244	0.248	0.248	0.248	0.248	0.248	0.248	0.248	0.248	0.248	0.248	0.248
0.252	0.252	0.252	0.252	0.251	0.257	0.253	0.252	0.252	0.252	0.252	0.252	0.252	0.252	0.252	0.252	0.252	0.252	0.252
0.251	0.251	0.251	0.251	0.252	0.249	0.255	0.249	0.251	0.251	0.251	0.251	0.251	0.251	0.251	0.251	0.251	0.251	0.251
0.249	0.249	0.249	0.249	0.247	0.247	0.245	0.254	0.249	0.249	0.249	0.249	0.249	0.249	0.249	0.249	0.249	0.249	0.249
0.246	0.246	0.246	0.246	0.246	0.246	0.246	0.246	0.251	0.244	0.239	0.241	0.246	0.246	0.246	0.246	0.246	0.246	0.246
0.252	0.252	0.252	0.252	0.252	0.252	0.252	0.252	0.252	0.257	0.252	0.253	0.252	0.252	0.252	0.252	0.252	0.252	0.252
0.251	0.251	0.251	0.251	0.251	0.251	0.251	0.251	0.248	0.249	0.257	0.250	0.251	0.251	0.251	0.251	0.251	0.251	0.251
0.251	0.251	0.251	0.251	0.251	0.251	0.251	0.251	0.248	0.250	0.252	0.256	0.251	0.251	0.251	0.251	0.251	0.251	0.251
0.332	0.332	0.332	0.332	0.332	0.332	0.332	0.332	0.332	0.332	0.332	0.332	0.341	0.325	0.326	0.332	0.332	0.332	0.332
0.333	0.333	0.333	0.333	0.333	0.333	0.333	0.333	0.333	0.333	0.333	0.333	0.326	0.341	0.330	0.333	0.333	0.333	0.333
0.335	0.335	0.335	0.335	0.335	0.335	0.335	0.335	0.335	0.335	0.335	0.335	0.333	0.334	0.344	0.335	0.335	0.335	0.335
0.250	0.250	0.250	0.250	0.250	0.250	0.250	0.250	0.250	0.250	0.250	0.250	0.250	0.250	0.250	0.254	0.247	0.248	0.249
0.249	0.249	0.249	0.249	0.249	0.249	0.249	0.249	0.249	0.249	0.249	0.249	0.249	0.249	0.249	0.249	0.254	0.245	0.244
0.249	0.249	0.249	0.249	0.249	0.249	0.249	0.249	0.249	0.249	0.249	0.249	0.249	0.249	0.249	0.246	0.248	0.254	0.249
0.252	0.252	0.252	0.252	0.252	0.252	0.252	0.252	0.252	0.252	0.252	0.252	0.252	0.252	0.252	0.252	0.250	0.253	0.257

**Table A2:** Weighted supermatrix.

0.049	0.049	0.049	0.049	0.049	0.049	0.049	0.049	0.049	0.049	0.049	0.049	0.049	0.049	0.049	0.049	0.049	0.049	0.049
0.049	0.049	0.049	0.049	0.049	0.049	0.049	0.049	0.049	0.049	0.049	0.049	0.049	0.049	0.049	0.049	0.049	0.049	0.049
0.048	0.048	0.048	0.048	0.048	0.048	0.048	0.048	0.048	0.048	0.048	0.048	0.048	0.048	0.048	0.048	0.048	0.048	0.048
0.049	0.049	0.049	0.049	0.049	0.049	0.049	0.049	0.049	0.049	0.049	0.049	0.049	0.049	0.049	0.049	0.049	0.049	0.049
0.054	0.054	0.054	0.054	0.054	0.054	0.054	0.054	0.054	0.054	0.054	0.054	0.054	0.054	0.054	0.054	0.054	0.054	0.054
0.055	0.055	0.055	0.055	0.055	0.055	0.055	0.055	0.055	0.055	0.055	0.055	0.055	0.055	0.055	0.055	0.055	0.055	0.055
0.054	0.054	0.054	0.054	0.054	0.054	0.054	0.054	0.054	0.054	0.054	0.054	0.054	0.054	0.054	0.054	0.054	0.054	0.054
0.054	0.054	0.054	0.054	0.054	0.054	0.054	0.054	0.054	0.054	0.054	0.054	0.054	0.054	0.054	0.054	0.054	0.054	0.054
0.049	0.049	0.049	0.049	0.049	0.049	0.049	0.049	0.049	0.049	0.049	0.049	0.049	0.049	0.049	0.049	0.049	0.049	0.049
0.051	0.051	0.051	0.051	0.051	0.051	0.051	0.051	0.051	0.051	0.051	0.051	0.051	0.051	0.051	0.051	0.051	0.051	0.051
0.050	0.050	0.050	0.050	0.050	0.050	0.050	0.050	0.050	0.050	0.050	0.050	0.050	0.050	0.050	0.050	0.050	0.050	0.050
0.050	0.050	0.050	0.050	0.050	0.050	0.050	0.050	0.050	0.050	0.050	0.050	0.050	0.050	0.050	0.050	0.050	0.050	0.050
0.068	0.068	0.068	0.068	0.068	0.068	0.068	0.068	0.068	0.068	0.068	0.068	0.068	0.068	0.068	0.068	0.068	0.068	0.068
0.069	0.069	0.069	0.069	0.069	0.069	0.069	0.069	0.069	0.069	0.069	0.069	0.069	0.069	0.069	0.069	0.069	0.069	0.069
0.069	0.069	0.069	0.069	0.069	0.069	0.069	0.069	0.069	0.069	0.069	0.069	0.069	0.069	0.069	0.069	0.069	0.069	0.069
0.045	0.045	0.045	0.045	0.045	0.045	0.045	0.045	0.045	0.045	0.045	0.045	0.045	0.045	0.045	0.045	0.045	0.045	0.045
0.045	0.045	0.045	0.045	0.045	0.045	0.045	0.045	0.045	0.045	0.045	0.045	0.045	0.045	0.045	0.045	0.045	0.045	0.045
0.045	0.045	0.045	0.045	0.045	0.045	0.045	0.045	0.045	0.045	0.045	0.045	0.045	0.045	0.045	0.045	0.045	0.045	0.045
0.046	0.046	0.046	0.046	0.046	0.046	0.046	0.046	0.046	0.046	0.046	0.046	0.046	0.046	0.046	0.046	0.046	0.046	0.046

## Appendix C. The VIKOR Results

**Table A3:** The results of VlseKriterijumska Optimizacija I Kompromisno Resenje (VIKOR).

Criteria	Weight	JV	TPLC	TPSC	JRD	Aspired	Worst	JV	TPLC	TPSC	JRD
*c*_11_	0.049	3.222	3.556	3.333	3.778	5.000	3.222	0.049	0.039	0.046	0.033
*c*_12_	0.049	4.000	3.667	4.111	3.333	5.000	3.333	0.030	0.039	0.026	0.049
*c*_13_	0.048	3.667	3.444	3.667	3.222	5.000	3.222	0.036	0.042	0.036	0.048
*c*_14_	0.049	2.889	3.667	3.889	3.667	5.000	2.889	0.049	0.031	0.026	0.031
*c*_21_	0.054	4.333	2.222	2.111	2.444	5.000	2.111	0.012	0.052	0.000	0.047
*c*_22_	0.055	3.667	4.444	4.111	3.778	5.000	3.667	0.055	0.023	0.036	0.050
*c*_23_	0.054	3.444	3.667	3.889	3.889	5.000	3.444	0.054	0.047	0.039	0.039
*c*_24_	0.054	4.111	4.222	4.333	4.000	5.000	4.000	0.048	0.042	0.036	0.054
*c*_31_	0.049	4.444	3.111	3.000	3.556	5.000	3.000	0.014	0.046	0.049	0.036
*c*_32_	0.051	3.778	3.111	3.222	3.667	5.000	3.111	0.033	0.051	0.048	0.036
*c*_33_	0.050	3.222	2.556	3.000	2.778	5.000	2.556	0.037	0.050	0.041	0.046
*c*_34_	0.050	3.889	3.222	3.444	3.889	5.000	3.222	0.031	0.050	0.044	0.031
*c*_41_	0.068	3.889	2.889	2.444	2.667	5.000	2.444	0.030	0.057	0.068	0.062
*c*_42_	0.069	3.222	3.000	2.333	2.667	5.000	2.333	0.046	0.051	0.069	0.060
*c*_43_	0.069	3.889	2.889	3.222	3.111	5.000	2.889	0.036	0.069	0.058	0.062
*c*_51_	0.045	3.222	4.111	4.111	4.111	5.000	3.222	0.045	0.023	0.023	0.023
*c*_52_	0.045	2.444	3.333	3.556	4.222	5.000	2.444	0.045	0.029	0.025	0.014
*c*_53_	0.045	3.111	2.667	2.667	3.000	5.000	2.667	0.037	0.045	0.045	0.039
*c*_54_	0.046	2.333	2.333	2.889	3.222	5.000	2.333	0.046	0.046	0.036	0.030
sum	1.000	66.778	62.111	63.333	65.000	Value of MV		0.000	1.000	0.580	0.560
*S*_k_								0.732	0.833	0.752	0.791
*Q*_k_								0.055	0.069	0.069	0.062
Rank								1	4	3	2

Remark: JV, “joint venture”; JRD, “joint R&D”; TPSC, “technology and patent sharing contracts”; TPLC, “technology and patent licensing contracts.”
